# Transcriptional Profiling and Identification of Heat-Responsive Genes in Perennial Ryegrass by RNA-Sequencing

**DOI:** 10.3389/fpls.2017.01032

**Published:** 2017-06-21

**Authors:** Kehua Wang, Yanrong Liu, Jinli Tian, Kunyong Huang, Tianran Shi, Xiaoxia Dai, Wanjun Zhang

**Affiliations:** ^1^Department of Grassland Science, China Agricultural UniversityBeijing, China; ^2^National Energy R&D Center for Biomass, China Agricultural UniversityBeijing, China

**Keywords:** transcriptional profiling, heat-responsive genes, HSPs, perennial ryegrass

## Abstract

Perennial ryegrass (*Lolium perenne*) is one of the most widely used forage and turf grasses in the world due to its desirable agronomic qualities. However, as a cool-season perennial grass species, high temperature is a major factor limiting its performance in warmer and transition regions. In this study, a *de novo* transcriptome was generated using a cDNA library constructed from perennial ryegrass leaves subjected to short-term heat stress treatment. Then the expression profiling and identification of perennial ryegrass heat response genes by digital gene expression analyses was performed. The goal of this work was to produce expression profiles of high temperature stress responsive genes in perennial ryegrass leaves and further identify the potentially important candidate genes with altered levels of transcript, such as those genes involved in transcriptional regulation, antioxidant responses, plant hormones and signal transduction, and cellular metabolism. The *de novo* assembly of perennial ryegrass transcriptome in this study obtained more total and annotated unigenes compared to previously published ones. Many DEGs identified were genes that are known to respond to heat stress in plants, including HSFs, HSPs, and antioxidant related genes. In the meanwhile, we also identified four gene candidates mainly involved in C_4_ carbon fixation, and one TOR gene. Their exact roles in plant heat stress response need to dissect further. This study would be important by providing the gene resources for improving heat stress tolerance in both perennial ryegrass and other cool-season perennial grass plants.

## Introduction

High temperature is a common abiotic stress for higher plants. It is estimated that the annual mean air temperature of about 23% of land on the earth is above 40°C (Leone et al., 2003). It could be getting worse due to current trends in global warming. It is anticipated the global temperature will increase another 1.7–3.8°C by 2100 (Wigley and Raper, 1992; IPCC, 2014). Temperature above the optimum for healthy plant growth is known as heat stress. Heat is understood as the upper temperature range in which active plant life is stressed, but is still possible (normally 10–15°C above the optimum temperature) (Schulze et al., 2005). Higher temperature affects the balance of growth and development by accelerating and redirecting metabolic processes. Under heat stress, plants respond at all levels, from morphological adaptations to physiological changes to molecular regulations. A number of important adaptive changes occur in terms of carbon and nitrogen metabolism, antioxidant responses, hormone homeostasis, and expression of many specific stress response genes/proteins (e.g., HSPs) (Schulze et al., 2005; Wahid et al., 2007).

Perennial ryegrass is native to Europe, Asia, and northern Africa, and becomes one of the most widely used forage and turf grasses worldwide. It has many desirable agronomic qualities, such as rapid establishment, long growing season, and high yield under favorable environments and conditions. However, as a cool-season perennial grasses, it grows best at the temperature range between 16 and 24°C, and the growth normally starts to decline when temperature exceeds 27°C. It does not withstand hot weather, and high temperature is a major factor limiting its performance in warmer and transition regions (Turgeon, 2011). Some studies of perennial ryegrass under heat stress have been conducted, but they are typically focused on breeding new cultivars, growth, or the physiological and biochemical aspects of the plants’ response (Minner et al., 1983; Sugita, 1991; Jiang and Huang, 2001; Kauffman et al., 2007; Zhang et al., 2013; Barnes et al., 2014; Chen et al., 2016; Wang and Xiong, 2016). To date, data regarding the molecular mechanism of perennial ryegrass responding to heat stress is very limited.

Transcriptomic studies have been widely adopted to systematically investigate the genes either involved in certain bioprocess and development stage or responses to different abiotic and biotic changes, particularly with the development of high-throughput next generation sequencing (NGS) (Wang et al., 2009; Hyun et al., 2012; Gao et al., 2013; Nejat et al., 2015). A wide spectrums of heat response genes have been identified in different plant species using transcriptomic methods, including *Arabidopsis* (Rizhsky et al., 2002; Larkindale and Vierling, 2008; Song et al., 2016), rice (*Oryza sativa*) (Sarkar et al., 2014; Wu et al., 2015), maize (*Zea mays*) (Dutra et al., 2015; Frey et al., 2015; Casaretto et al., 2016), tomato (*Solanum lycopersicum*) (Bita et al., 2011; Cheng et al., 2012), potato (*Solanum tuberosum*) (Ginzberg et al., 2009; Tang et al., 2016), carnation (*Dianthus caryophyllus*) (Wan et al., 2015), wheat (*Triticum aestivum*) (Qin et al., 2008), and *Brachypodium distachyon* (Chen and Li, 2017). However, little information is available regarding heat responsive genes at global transcriptome level in the perennial cool-season grasses, especially perennial ryegrass. Only very recently, Wang Y. et al. (2016) analyzed perennial ryegrass under temperature stress by RNA-Seq, and they mainly focused on heat shock factor (*HSF*) genes. There are many more other genes are important for plants in response to heat stress, such as heat shock proteins (HSPs), reactive oxygen species (ROS) scavenger genes, signal transduction and other transcription factors (TFs) (Schulze et al., 2005; Kotak et al., 2007; Wahid et al., 2007; Hasanuzzaman et al., 2013).

In this study, we generated a *de novo* transcriptome using a leaf cDNA library of perennial ryegrass subjected to short-term heat stress. Then the expression profiling and identification of perennial ryegrass heat response genes by digital gene expression (DGE) analyses was performed using this transcriptome as the reference set of sequences. The objectives of this study were to identify gene candidates with changed transcript levels in perennial ryegrass leaves under heat stress, particularly, those genes potentially involved in transcriptional regulation, antioxidant responses, plant hormones and signal transduction, and cellular metabolism. This study would be important by providing the gene resources for improving heat stress tolerance in both perennial ryegrass and other cool-season perennial grass plants.

## Materials and Methods

### Plant Materials and Heat Stress Treatment

Perennial ryegrass ‘Citation Fore’ (PureSeed, Canby, OR, United States) was used in this study. Grass plants were grown in a greenhouse at China Agricultural University (Beijing, China) at 25 ± 3/18 ± 2°C (day/night) for 14 h (day) and 10 h (night) before moving into growth chambers for high temperature treatments. Plants were clonally propagated from tillers and grown in plastic pots (12.0 cm × 10.5 cm) using a soil mixture of silica sand and peat (1:1, v/v). Plants were cut weekly to maintain uniform above-ground growth, watered as needed to avoid water stress, and fertilized weekly with Miracle-Gro TEP6 (24-12-14, N-P-K; Scotts, Wuhan, China) at 5 kg N ha^-1^.

Four months after been propagated from a single tiller, grasses were moved to a growth chamber for a 1-week adaptation. The growth chamber was set as the following: relative humidity 70%/85% (day/night), 22/16°C, and a 14-h photoperiod with 400 μmol s^-1^ m^-2^ PAR (photosynthetically active radiation). A week later, half of the adapted grasses were switched into another growth chamber (same model) for a short-term high temperature treatment (6 h, 35/35°C). Leaf tissues were harvested, immediately frozen with liquid nitrogen, and then stored at -80°C until analysis.

### RNA Isolation and Illumina Sequencing

Total RNA was isolated from the perennial ryegrass leaf samples with Trizol reagent (Invitrogen, Carlsbad, CA, United States). The purity and integrity of the RNA were evaluated using the Implen Nano-Photometer^®^ N50 (München, Germany) and Agilent 2100 Bioanalyzer (United States), respectively. The concentration of the RNA was determined using Qubit^®^ 2.0 Fluorometer. A total of 1.5 μg RNA each sample was used for the RNA-seq analysis. NEBNext^®^ Ultra^TM^ RNA Library Prep Kit for Illumina^®^ (NEB, United States) was used to generate the sequencing libraries. The cDNA (150∼300 bp) were purified selectively from the libraries using AMPure XP system (Beckman Coulter, Beverly, MA, United States). PCR Enriched cDNAs were used to create the final cDNA library, and then sequenced with Illumina HiSeq^TM^ 2500 platform, using paired-end reads (2 × 100 nucleotides). The sequencing was carried out at Novogene Corporation (Beijing, China).

### Real-Time Quantitative PCR Analysis for RNA-Seq Data Validation

Real-time quantitative PCR of 16 different genes using 7500 Real-Time PCR System (Applied Biosystems) was carried out to validate the RNA-seq results. Primer sequences for qRT-PCR were designed using Primer premier 6 software and were listed in Supplementary Table S1. Gene expression levels were calculated by the 2^-ΔΔCt^ method (Livak and Schmittgen, 2001). Each plate was repeated three times in independent runs for all reference and selected genes.

### Data Analysis

#### Quality Control

Clean reads/data were obtained from raw data after removing the reads containing adapter or ploy-N and other low-quality reads. In the meanwhile, the clean data Q20, Q30 value, GC-contents, and the level of sequence duplication were calculated (Supplementary Table S2).

#### Sequence Assembly and Gene Functional Annotation

Since there is no publicly available genome of perennial ryegrass (Pfeifer et al., 2013), Trinity was used to construct and accomplish the *de novo* assembly of the transcriptome here (Grabherr et al., 2011). Unigenes were blasted using blastx against databases publicly available, including Nr (NCBI non-redundant protein database^1^), Nt (NCBI non-redundant nucleotide sequences^1^), Pfam (Protein family), Swiss-Prot^2^, KEGG (the Kyoto Encyclopedia of Genes and Genomes pathway database^3^), KOG/COG (Cluster of Orthologous Groups database^4^), and GO (Gene Ontology), and the best aligning results were used to decide the direction of the sequence and CDS (coding sequence) of unigenes. A typical cutoff value of *E* < 10^-5^ was used. ESTScan (Iseli et al., 1999) was used to predict a unigene’s coding regions as well as to decide its sequence direction when it was found not to be aligned to any of the databases above.

#### Differential Expression Analysis

RSEM (Li and Dewey, 2011) was used to calculate gene expression levels. To further reveal the heat stress responsive genes, we performed comparative transcriptomic analysis among the pools of control and high temperature RNA samples. The genes with a *p*_adj_ (*P*-value-adjusted) < 0.05 were identified as differentially expressed (down- or up-regulated) genes (DEGs) according to (Anders and Huber, 2010). All the DEGs were further annotated by GO and KEGG pathway enrichment analyses. GO enrichment analysis of the DEGs was implemented by the GOseq R packages based Wallenius non-central hyper-geometric distribution (Young et al., 2010). The KOBAS software was performed to test the statistical enrichment of differential expression genes in KEGG pathways (Mao et al., 2005; Kanehisa et al., 2008).

## Results

### Illumina Paired-End Sequencing and Assembly

Here the library was sequenced according to Illumina paired-end protocol. After removing adaptors and low-quality reads, 62,723,918 clean paired-end reads (94.57% of the raw reads data) were obtained. And the average sample GC-rich content and the Q20 level was 55.53 and 95.21%, respectively. *De novo* assembly of the clean reads data with Trinity identified 290,039 contigs. The N50 gene size, average contig length, and the maximum contig length was 1920, 1023, and 16,826 bp, respectively. A total of 185,671 unigenes were obtained, and the longest length transcript for each unigene was selected for further analysis. The average length of unigenes was 675 bp, and transcripts with lengths of equal or greater than 500 bp accounted for about 51.5% of all transcripts (**Table 1**). The accession number of the transcriptome data deposited to Sequence Read Archive (SRA) is SUB2445006.

**Table 1:** Summary of sequencing and *de novo* assembly.

Item	Values
Clean reads	62,723,918
Clean bases (nt)	10,100,331,424
GC-content (%)	55.53
Q20 percentage (%)	95.21
Total assemble size (nt)	296,742,727
Number of contigs	290,039
Average length of contig (nt)	1,023
Shortest length of contig (nt)	201
Longest length of contig (nt)	16,826
N50	1,920
Total number of unigenes	185,671
Length of all unigenes (nt)	125,318,772
Average sequence size of unigenes (nt)	675
N50	1,169


### Annotation and Functional Classification

All assembled unigenes were submitted to a Blastx search against the public protein databases in order to validate and annotate the assembled unigenes. Among the total 185,671 unigenes, 96,106 (51.76%), 78,959 (42.52%), and 74,188 (39.95%) unigenes showed homology with the sequences in the databases of Nt, Nr, and SwissProt, respectively. And 121,271 unigenes were annotated in at least one database searched against (Supplementary Table S3).

**FIGURE 1:**
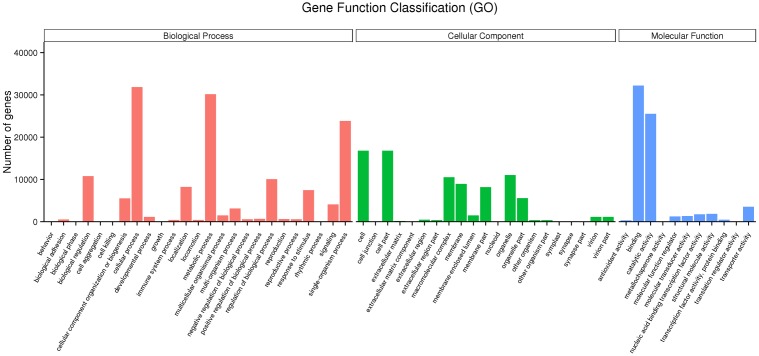
Gene ontology (GO) annotations of non-redundant consensus sequences of perennial ryegrass. Best hits were aligned to the GO database, and most consensus sequences were grouped into three major functional categories namely, biological process (BP), cellular component (CC), and molecular function (MF).

The unigene sequences were further characterized by the assignment of GO terms (**Figure 1** and Supplementary Table S4). In total, 1,961 functional GO terms were assigned among 60,117 unigenes with BLAST matching to known proteins. The most highly represented GO categories of biological processes were cellular processes (32,219 unigenes), metabolic processes (30,197 unigenes), and biological regulation (10,784 unigenes), which suggested a high degree of basic metabolic activity and biological regulation in the heat stressed tissues. Similarly, for the categories of cellular component, cell (16,786) and cell part (16,783) were the two mostly represented. Under the classification of molecular functions, the binding (32,219 unigenes) and catalytic activities (25,533) represented the two largest categories. Those GO categories of TFs (484 unigenes), signal transduction (4,112 unigenes), response to stimulus (7,467 unigenes), and antioxidant activity (318 unigenes) were important in general stress response, but with less assigned unigenes.

**FIGURE 2:**
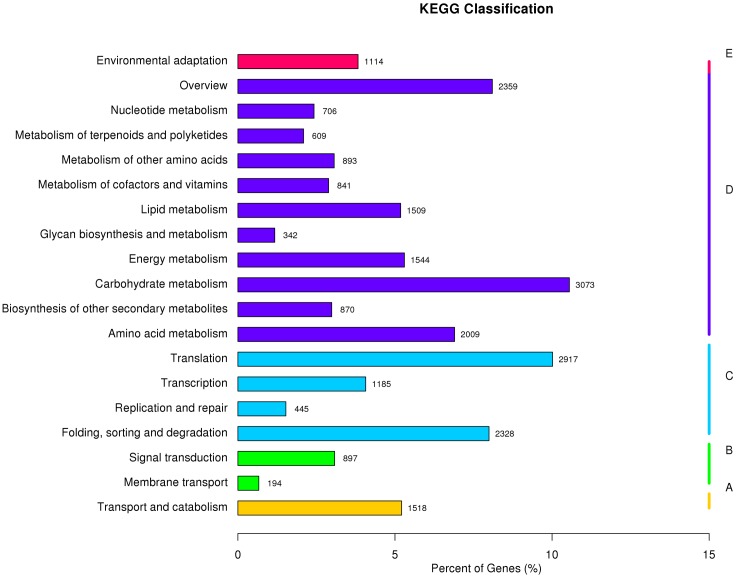
Pathway assignment of perennial ryegrass genes based on the Kyoto Encyclopedia of Genes and Genomes (KEGG) database. **(A)** Classification based on cellular process categories, **(B)** classification based on environmental information processing categories, **(C)** classification based on genetic information processing categories, **(D)** classification based on metabolism categories, and **(E)** classification based on organismal systems categories.

All unigenes were further aligned to the eukaryotic Ortholog Groups (KOG) database to predict and classify their possible functions. A total of 31,021 sequences were assigned to KOG classification of 26 categories, respectively. Based on the KOG classification, the unigenes were then analyzed using the KEGG pathway database. Out of the 185,671 unigenes identified in the study, 29,124 (15.69%) were assigned to132 KEGG pathways belonging to five main categories (**Figure 2** and Supplementary Table S5). Among the 132 KEGG pathways, 10 most assigned ones were carbohydrate metabolism (10.6%), translation (10%), overview (8.1%), folding, sorting and degradation (8%), amino acid metabolism (6.9%), energy metabolism (5.3%), transport and catabolism (5.2%), lipid metabolism (5.2%), transcription (4.1%), and environmental adaptation (3.8%). These results indicated that both active metabolic processes and environmental adaptation responses were occurring in perennial ryegrass.

### Differential Gene Expression of Perennial Ryegrass in Response to Heat Stress

**FIGURE 3:**
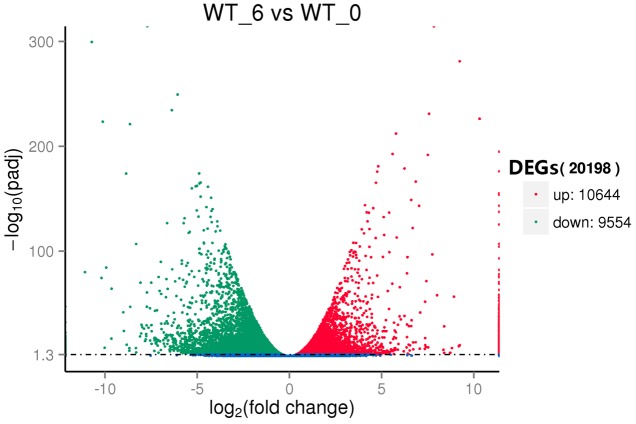
Volcano plot of the differentially expressed genes (DEGs) between the control (WT_0) and heat stressed (WT_6) perennial ryegrass.

To reveal the molecular events and identify genes with altered expression levels under heat stress, the DGE libraries were constructed using RNA from the pools of control and the heat stressed plant RNA samples and sequenced. Using the criteria of twofold up- or down-regulation [Log_2_FC (fold change) ≦ 1 or ≧ -1], 11,275 genes were identified as differentially-regulated genes (DEGs), including 4756 (42.2%) up-regulated and 6519 (57.8%) down-regulated unigenes (**Figure 3**). Strongly up-related genes (Log_2_FC ≦ 4) (3052) and down-related genes (Log_2_FC ≧ -4) (1132) under heat stress were further identified from expression profile analysis (Supplementary Table S6). These genes mostly comprised stress response genes, including HSPs, signal transduction factors, and TFs. Fifty-two HSP genes whose expressions were present under heat stress, but not in control. Interestingly, there were 99 highly induced genes (256-fold or higher) by heat stress whose functions were not known.

### Gene Ontology Analysis of the Functional Annotation and Classification of the DEGs

**FIGURE 4:**
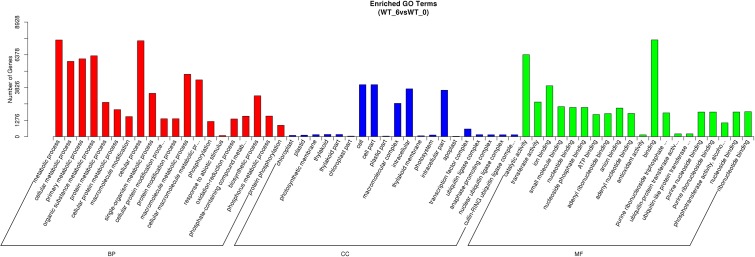
Gene ontology classifications of DEGs between the control and heat stressed perennial ryegrass. The *Y*-axis represents the number of DEGs in a category. The results of heat stressed (WT_6) vs. control (WT_0) are summarized in three main categories: BP, CC, and MF.

Gene Ontology classification of the significantly regulated genes was carried out to identify the heat stress response related functional processes in perennial ryegrass leaves. Among the total 251 identified sub-classifications of GO functions, the predominant 60 GO classifications were shown in **Figure 4**. Other than the commonly enriched GO classifications, such as metabolic process, cellular process, catalytic activity, both response to abiotic stimulus (GO:0009628) and antioxidant activity (GO:0016209) were among the predominantly enriched groups. In addition, regulated genes mainly related to temperature stress responses are also enriched, such as response to heat (GO:0009408), HSP binding (GO:0031072), and response to temperature stimulus (GO:0009266).

### KEGG Pathway Analysis of the Heat Responsive Genes

**FIGURE 5:**
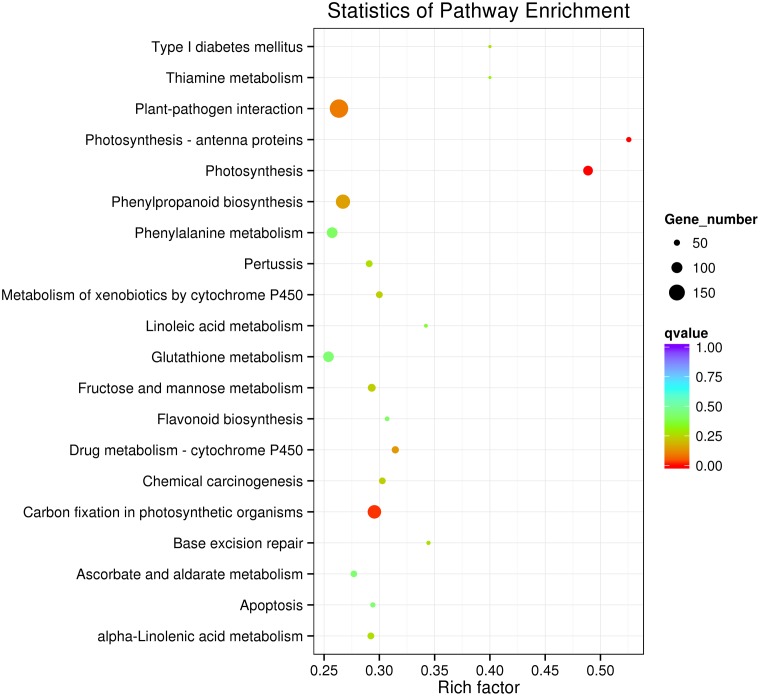
Kyoto Encyclopedia of Genes and Genomes enrichments of the annotated DEGs between the control and heat stressed perennial ryegrass. The left *Y*-axis indicates the KEGG pathway. The *X*-axis indicates the Rich factor. A high *q*-value is represented by blue, and a low *q*-value is represented by red.

**FIGURE 6:**
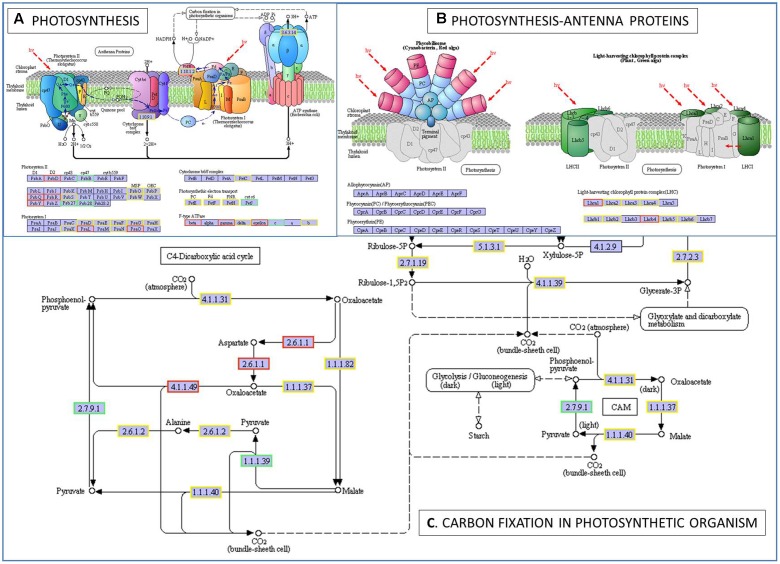
The pathways of photosynthesis **(A)**, photosynthesis-antenna proteins **(B)**, and carbon fixation in photosynthetic organism **(C)** enriched by KEGG analysis. A gene/protein name in a red and blue box represent up-regulation and down-regulation under heat stress, respectively. PsbD, photosystem II P680 reaction center D2 protein (EC:1.10.3.9); PsbB, photosystem II CP47 chlorophyll apoprotein; PsbQ, photosystem II oxygen-evolving enhancer protein 3; PsbR, photosystem II 10 kDa protein; Psb27, photosystem II Psb27 protein; Psb28, photosystem II 13 kDa protein; PsaL, photosystem I subunit XI; PsaO, photosystem I subunit; petJ, cytochrome c6; Lhca1, light-harvesting complex I chlorophyll a/b binding protein 1; Lhcb4, light-harvesting complex II chlorophyll a/b binding protein 4; EC:2.6.1.1, aspartate aminotransferase, cytoplasmic; EC:4.1.1.49, phosphoenolpyruvate carboxykinase (ATP); EC:2.7.9.1, pyruvate, orthophosphate dikinase; EC:1.1.1.39, malate dehydrogenase (decarboxylating).

To further determine whether the heat responsive genes were involved in specific pathways, the DEGs were used as objects to search against the KEGG pathway database. The top 20 obviously enriched pathways are shown in **Figure 5**. The ‘plant–pathogen interaction’ pathway enriched the most DEGs, but ‘photosynthesis – antenna proteins,’ ‘photosynthesis,’ and ‘carbon fixation in photosynthetic organisms’ were the most significantly enriched according to the adjusted *P*-values (**Figures 5**, **6**). For instance, in ‘photosynthesis’ group, three photosystem II genes, PsbD (photosystem II P680 reaction center D2 protein), PsbQ (photosystem II oxygen-evolving enhancer protein 3), and PsbR (photosystem II 10 kDa protein) were up-regulated, while two other photosystem II genes (Psb27, photosystem II Psb27 protein; Psb28, photosystem II 13 kDa protein) and PetJ (cytochrome c6) in photosynthetic electron transport were down-regulated. In ‘photosynthesis – antenna proteins’ group, Lbca1 (light-harvesting complex I chlorophyll a/b binding protein 1) and Lbcb4 (light-harvesting complex II chlorophyll a/b binding protein 4) were stimulated. For ‘carbon fixation’ group, aspartate aminotransferase (AST), cytoplasmic and phosphoenolpyruvate carboxykinase (ATP) (PEPCK) were up-regulated, while pyruvate orthophosphate dikinase (PPDK) and malate dehydrogenase (decarboxylating) (NAD-ME) were down-regulated. Several other less enriched but important pathways for heat responses included plant hormone signal transduction, zeatin biosynthesis, and peroxisome (Supplementary Table S7 and **Figure S1**). Interestingly, mTOR signaling pathway, a signaling network mostly known for its role in a number of human pathological conditions (Hall, 2008), was identified by KEGG analysis as well (**Supplementary Figure S2**).

### HSPs in Response to Heat Stress

Out of the list of significantly regulated genes, 63 HSPs were largely up-regulated (Log_2_FC ≧ 4) and 1 HSP was highly down-regulated (Log_2_FC ≦ -4). Of these highly up-regulated HSP genes, 18 genes were various types of small HSP (sHSP) (cytosolic classes I, II, and III, chloroplast, mitochondrial and endoplasmic reticulum), 6 coded for HSP60, 16 for HSP70, 15 for HSP90, and 8 for HSP101. The highly down-regulated HSP belonged to HSP70 family (Supplementary Tables S6, S8).

### Transcription Factors in Response to Heat Stress

Transcription factors of different families were significantly regulated in response to heat stress. HSF, AP2/EREBP, MYB, bHLH, and DIVARICATA families were among the strongly up- or down-regulated TFs (Log_2_FC ≧ 2/≦ -2). In addition, one gene from HSF (c37944_g1, *Hsf*24-like), AP2/EREBP (c25197_g1, ERF003), NAC (c11267_g1, NAC029), DIVARICATA (c1152_g1, TF DIVARICATA), and MBF1 (c85706_g1, MBF1a) family was only found after heat stress. For HSFs, 10 of them were significantly up-regulated, including the one present only under heat and one increased over 21-folds (c37944_g1, *Hsf24*; c61648_g4, *HsfA*-2a). In contrast, 11 *HSF* genes exhibited lower expression levels compared to those under the control condition, and *HsfA*-2d was the mostly strongly down-regulated (Log_2_FC values of -4.0) (Supplementary Tables S6, S8).

### Effects of Heat Stress on Antioxidant Response Genes

The group of antioxidant activity (GO:0016209) was enriched by GO term analysis. Over 130 genes involved in antioxidant response were either up- or down-regulated. *SODA* (mitochondrial Fe/Mn SOD), *SODB* (chloroplastic Fe/Mn SOD), and *SODCP* (chloroplastic Cu/Zn SOD) were all up-regulated. Most of the 24 peroxidase (*POD)* genes identified (peroxidase 1, 2, 3, 4, 5, 12, 15, 16, 17, 18, 21, 24, 35, 42, 43, 47, 51, 54, 56, 65, 68, 70, N1, and P7) were down-regulated, except peroxidase 12, 21, 42, 51, and N1 (*PER12*, *PER21*, *PER42*, *PER51*, and *poxN1*). In contrast, the majority of the differentially expressed ascorbate *POD* genes (*APX1*, *APX2*, *APX3*, *APX4*, *APX6*, and *APXT*) were up-regulated, except for *APX4* and a copy of *APX2*. Both of them were slightly down-regulated (Log_2_FC was about -0.5). Similarly, catalase (*CAT*) genes were mostly up-regulated, such as *CAT1* and *CAT2*. Respiratory burst oxidase genes (*RBOH)* were mainly down-regulated, including *RBOHB*, *RBOHC*, *RBOHE*, and *RBOHF* (Supplementary Tables S6, S8).

### RT-qPCR Validation of Gene Expression Profiles

RT-qPCR was carried out for 16 randomly selected DEGs. Histograms were produced by comparing the FPKM determined by transcriptome analysis and RT-qPCR. Expression quantities of the selected genes using RT-qPCR were consistent with the results obtained with RNA-Seq analysis (*R*^2^ = 0.874, *P* < 0.01), indicating reproducibility and credible RNA-seq data (**Supplementary Figures S3**, **S4**). One exception was c63520_g2. Its expression nearly unchanged under heat stress by RT-qPCR, but increased about twofolds in RNA-Seq DEG analysis.

## Discussion

Transcriptome analysis of perennial ryegrass was firstly reported nearly 10 years ago employing a Serial Analysis of Gene Expression method (SAGE), which revealed 2772 transcripts (Sathish et al., 2007). Later Studer et al. (2012) acquired 9399 non-redundant contigs and singletons of perennial ryegrass from 25,744 high-quality EST reads generated by Sanger and 454 sequencing. More recently, *de novo* assembly of the perennial ryegrass transcriptome using the RNA-seq strategy resulted in 185,833 transcripts with an average length of 830 bps, and 50,860 transcripts (27.38%) were functionally annotated (Farrell et al., 2014). A very recent study (Wang Y. et al., 2016) analyzed perennial ryegrass under temperature stress by RNA-Seq, and they generated a total of 73,125 unigenes with an average length of 723 bps. Moreover, they found a total of 20,183 DEGs, including 15,036 up-regulated and 5147 down-regulated DEGs. Here our transcriptome assembly resulted in 185,671 unigenes (29,0039 transcripts), and 121,271 unigenes (65.32% of all unigenes) were annotated (**Table 1**). In addition, total 20198 DEGs (10.9% of all unigenes) were detected, which consisted of 10644 up-regulated and 9554 down-regulated DEGs (**Figure 3**). More total unigenes and annotated unigenes were detected when compared to previously published ones, which suggested that the data obtained here were comparable or larger and sufficient for further analyzing and mining genes expressing differentially in perennial ryegrass under heat stress.

In higher plants, heat stress redirects protein synthesis by decreasing the transcription and translation of normal proteins, and stimulating the synthesis of a new set of proteins: HSPs (Schulze et al., 2005). HSPs function mainly as molecular chaperones that help other proteins maintain their native conformation, thus improving protein stability under stresses (Wahid et al., 2007). Based on their approximate molecular weight, the principal HSPs are grouped into six conserved classes: HSP100/Clp, HSP90/HtpG, HSP70/DnaK, HSP60/GroEl, HSP40/DnaJ, and the small heat shock proteins (sHSPs) (Bharti and Nover, 2002; Schulze et al., 2005). The up-regulation of this gene group is well documented when cells are exposed to elevated temperatures or other stresses (Ada et al., 1987; Lee et al., 2000; Agarwal et al., 2003; Kant et al., 2008). It is therefore not surprising that the expression of many HSPs increased after exposure to heat stress in the study. And 86 of them are strongly up-regulated, including 19 sHSP, 21 HSP40, 6 HSP60, 16 HSP70, 15 HSP90, and 9 HSP101 genes (Supplementary Table S8). Low molecular weight HSPs or sHSPs are the most dominant proteins produced in higher plants subjected to heat stress (Waters et al., 1996; Sun et al., 2002). SHSPs in the study here were the most abundant HSPs regarding the read counts. For example, one annotated sHSP (chloroplast low molecular weight HSP *HSP26.7b*, c56087_91) increased 584 times after heat stress, from 37.4 to 21833.4 (read counts). Wang and Luthe (2003) found that several chloroplast (CP) *HSP26* genes were up-regulated upon heat stress, and suggested that quantitative differences of total CP-sHSP are more critical in conferring enhanced thermo-tolerance of creeping bentgrass (*Agrostis stolonifera*). Li Z. et al. (2013) reported that the expression of *ApHSP16.5* and *ApHSP26.8* was induced much more pronounced in the *OsSIZ1* transgenic plants compared to that in WT controls, and transgenic plants were more heat tolerant than WT ones. Many studies have reported that plant heat stress tolerance is positively correlated with differences in CP-sHSP levels (Downs et al., 1998; Preczewski et al., 2000; Knight and Ackerly, 2001; Li Z. et al., 2013; Wang K. et al., 2016). All the nine *HSP100* genes were only present under heat stress, indicating their important role in plant thermo-tolerance. In maize (*Z. mays* L.) and *Arabidopsis*, HSP100 are thought to be causally involved in the capacity to acquire heat stress tolerance (Hong and Vierling, 2001; Nieto-Sotelo et al., 2002). Meanwhile, three *HSP40* and one *HSP70* (c51623_g5) were strongly down-regulated. Wang Y. et al. (2016) and Hu et al. (2014) reported similar HSP changes in perennial ryegrass and/or tall fescue using transcriptome analysis, but with fewer *HSP* genes.

The molecular mechanism leading to HSP expression under stresses are not entirely understood, but HSFs serve as the terminal components of signal transduction mediating the expression of HSPs and other heat stress induced transcripts are widely accepted (Pockley, 2003; Kotak et al., 2007; Penfield, 2008). Plants possess multiple HSF-encoding genes, with 19 members defined in castor bean (*Ricinus communis*), 21 in *Arabidopsis*, 24 in *B. distachyon* and millet (*Setaria italica*), 25 in rice, 27 in tomato, 30 in poplar (*Populus trichocarpa*) and maize, 40 in cotton (*Gossypium raimondii*), and 56 in wheat (Guo et al., 2008; Scharf et al., 2012; Xue et al., 2014). Here we found 39 *HSF* unigenes, and further gene sequence comparison identified 33 members of HSFs (Supplementary Table S8). Wang Y. et al. (2016) reported 52 *HSF*s in perennial ryegrass and 74 *HSF*s in tall fescue (*Festuca arundinacea*) transcriptomes, which are larger than most known species, and also more than what we identified here. HsfA1a, HsfA2, and HsfB1 was found to form a regulatory network in tomato that regulates the expression of HS-responsive genes, and HsfA2 was thought to be the major HSF in thermo-tolerant cells (Mishra et al., 2002; Baniwal et al., 2004; Guo et al., 2016). Whereas, analysis of *Arabidopsis HsfA1a*, *HsfA1b*, and *HsfA2* knockout mutants indicates that *HsfA1a* and *HsfA1b* are vital in the early phase of heat shock responsive gene expression, and that *HsfA2* controls gene expression under long-term heat stress and recovery condition after stress (Lohmann et al., 2004; Schramm et al., 2006; Kotak et al., 2007). Here we found members of class *HsfA2* were highest in quantity among all of the Hsf, and most of the *HsfA2* genes *(HsfA2a, b*, and *c*) were up-regulated under heat stress, except *HsfA2d*. A similar finding was reported in both tall fescue and perennial ryegrass (Wang Y. et al., 2016). However, here *HsfA1* in perennial ryegrass was largely unchanged. Wang Y. et al. (2016) reported *HsfA1* was weakly up-regulated or even down-regulated in perennial ryegrass or tall fescue. And they postulated that *HsfA2* and *HsfA1*in tall fescue and perennial ryegrass function independently to enhance thermo-tolerance. Likewise, heat stress also resulted in a significant increase in the expressions of other *HSF*s, such as *HsfA3*, which was consistent to results observed in other studies (Hu et al., 2014; Xue et al., 2014; Wang Y. et al., 2016).

The expression of *HSF*s triggers changes in expression of downstream target genes (Kotak et al., 2007). Except the major target genes, HSPs as discussed above, *HSF*s were also found to directly regulate several other genes in *Arabidopsis* including metabolic enzymes such as inositol-3-phosphate synthase2 (*Ips2*) and galactinol synthase 1 (*GolS1*) (Panikulangara et al., 2004; Nishizawa et al., 2006), and an enzyme in antioxidant response network, *APX2* (ascorbate peroxidase) (Miller and Mittler, 2006; Nishizawa-Yokoi et al., 2009). After specifically investigating the expression of the three genes under heat stress, we found the expression of *APX*s and *IPS2* increased significantly upon heat stress, but *GOLS* decreased under heat stress, which is consistent with a previous study on perennial ryegrass and tall fescue under heat stress (Wang Y. et al., 2016). These indicated that there might be some difference in *HSF* regulation between monocots (grasses) and dicots (*Arabidopsis*). APX is one of the major H_2_O_2_-reducing peroxidases and is important for antioxidant response in plants (Blokhina et al., 2003; Ishikawa and Shigeoka, 2008). The expression of the *APX* gene family in *Arabidopsis* is regulated by heat stress, which suggests a link links between heat stress response and oxidative stress (Panchuk et al., 2002; Suzuki et al., 2013; Wan et al., 2015).

Heat stress can induce oxidative stress. For instance, generation of ROS including singlet oxygen (^1^O_2_), superoxide radical (O^2-^), hydrogen peroxide (H_2_O_2_), and hydroxyl radical (OH^-^) are thought to be symptoms of cellular injury because of heat stress (Liu and Huang, 2000; Larkindale and Huang, 2004; Wang et al., 2012). Hsfs have been suggested to function as molecular sensors that directly sense and respond to the signals of ROS, which in turn activate the HSP expression and anti-oxidative genes. And HSF-binding motifs in the promoter region of genes associated with the ROS gene network have been detected (Miller and Mittler, 2006). Under heat stress the antioxidant enzyme system of plants is enhanced in response to increased ROS levels (Miller et al., 2008; Choudhury et al., 2013). In study of carnation (*Dianthus caryophyllus*) after short-term high temperature stress by RNA-seq, several genes encoding antioxidant enzymes including, *APX*s, *AOX*, thioredoxin, and glutathione *S*-transferase (*GST*) showed an increase in expressions (Wan et al., 2015). In the study herein, other than *APX*s and thioredoxin, we also identified several other genes involved in antioxidant responses, such as superoxide dismutases (*SOD*), *CAT*, *POD*, glutathione peroxidases (*GPX*), and *RBOH* proteins. Most of the genes were up-regulated, except some *POD* genes and *RBOH* genes (Supplementary Table S8). SOD is the first line of defense against ROS, and is the key enzyme to scavenge O_2_^-^ produced in the cells by catalyzing superoxide dismutation into hydrogen peroxide and oxygen. There are three different types of SOD isoforms according to their metal cofactors, namely Mn-SOD in mitochondria, Fe-SOD in chloroplasts, and Cu/Zn-SOD in cytosol and chloroplasts (Blokhina et al., 2003; Wahid et al., 2007). Here the expression levels of three mitochondrial *Mn-SOD*s, three chloroplastic *Fe-SOD*s, and one chloroplastic *Cu/Zn-SOD* were found to be increased at various degrees. *SOD* is known to be induced in different plants under heat stress (Larkindale and Huang, 2004). In plants, H_2_O_2_ is finely regulated by CAT and a group of peroxidases localized in nearly all compartments of plant cells, including APX and GPX (Blokhina et al., 2003; Ishikawa and Shigeoka, 2008; Gill and Tuteja, 2010). Catalase is indispensable for ROS detoxification during stressed conditions, and it is important in removing H_2_O_2_ generated in peroxisomes by oxidases participating in b-oxidation of fatty acids, purine catabolism and photorespiration (Garg and Manchanda, 2009; Saed-Moucheshi et al., 2014). Heat stress usually stimulates both the CAT activity and gene expression (Sairam et al., 2000; Lin et al., 2010). Here we found both *CAT1* and *CAT2* were up-regulated in response to heat stress. GPX are a family of diverse isozymes using GSH to reduce lipid hydroperoxides and H_2_O_2_, and therefore protect plant cells from oxidative stress (Noctor et al., 2002). In *Arabidopsis*, there are seven *GPX* in cytosol, chloroplast, mitochondria and endoplasmic reticulum, named *AtGPX1-AtGPX7* (Millar et al., 2003). In the study here, five *GPX*s were significantly up-regulated, and one was down-regulated. Thioredoxins are a class of small redox proteins known to play important roles in redox signaling and other biological processes (Arner and Holmgren, 2000; Dos Santos and Rey, 2006). Our study found a total of 18 unigenes encoding thioredoxin or thioredoxin-like proteins. Among of all the 18 thioredoxins, 13 of them were induced by heat stress. This result indicated thioredoxins could be one of the dominant antioxidants present in plants during heat shock, which is in agreement with previous studies in tobacco (*Nicotiana tabacum*) and carnation (Rizhsky et al., 2002; Wan et al., 2015).

Respiratory burst oxidase homologs gene family encode NADPH oxidase that generate super oxide and function in ROS promoted stress reactions and signaling (Jiang et al., 2011; Demidchik, 2015). Available data suggest that NADPH oxidase activity is required during stress primarily for the following four physiological and biochemical functions: (1) adjusting gene expression and metabolism by recognizing stress factor and its intensity; (2) stomatal closure under drought; (3) triggering the programed cell death; and (4) central “processor” of the signals of stress, defense and development (Torres and Dangl, 2005; Demidchik, 2015). There are 10 genes in *Arabidopsis* (*AtRBOH A-J*) and 9 in rice. It exists in other plant species as well (Torres and Dangl, 2005; Kawahara et al., 2007). Here six unigenes were identified as *RBOH*, and their expressions were mainly down-regulated, indicating a reduced ROS production after 6 h heat stress. Reactive oxygen species may act as signal molecules for plant growth and development, but excessive ROS are detrimental and can cause the autocatalytic peroxidation of pigments and membrane lipids, which leads to the loss of semi-permeability of membranes and modifies their functions (Senthil-Kumar et al., 2007; Wahid et al., 2007). Owing to the biological paradox, ROS levels are normally well regulated by their generation rate and the degradation rate as affected by the ROS scavenging capacity of antioxidant enzymes and antioxidants (Asada, 1999; Blokhina et al., 2003). In this respect, the changes of antioxidant related genes under heat stress showed a systematic response to maintain cellular homeostasis of ROS.

Photosynthesis known to be sensitive to environment stresses, and heat stress affect plant photosynthesis negatively (Mathur et al., 2014). For instance, 1 day high temperature at 35°C decreased canopy photosynthesis of creeping bentgrass (Xu and Huang, 2000). Photochemical reactions in thylakoid lamellae and carbon metabolism in chloroplast stroma have been indicated as the major injury sites under heat stress (Wahid et al., 2007). Here KEGG pathway analysis revealed that ‘photosynthesis,’ ‘photosynthesis – antenna proteins,’ and ‘carbon fixation in photosynthetic organisms’ were the most significantly enriched groups (**Figures 5**, **6**).

The photosystem II (PSII) reaction center core consists of two chlorophyll binding proteins, D1 and D2, which are encoded by chloroplast *PsbA* and *PsbD* genes, respectively (Thum et al., 2001; Stroch et al., 2004; Armbruster et al., 2010). The D2 protein is needed for the assembly of a stable PSII complex. And it generates non-radiative energy dissipation, which is a very effective mechanism to protect the PSII from photodamage (Stroch et al., 2004; Song et al., 2014). Sane et al. (2002) suggested that D2 protein accumulation may promote resistance to high excitation stress induced by exposure to either high light or low temperature. Here we observed an up-regulation of *PsbD* after heat stress, but not *PsbA*. Song et al. (2014) also reported a significant up-regulation of *PsbD* at 6 h of heat treatment. The result suggested that *PsbD* might be associated with mechanisms protecting against photodamage of perennial ryegrass under the stress condition.

Photosystem II inactivation by heat may be followed by dissociation of LHC II (light-harvesting complex II) (Mathur et al., 2014). The light-harvesting chlorophyll a/b binding proteins (LHCB and LHCA) are the apoproteins of the PSII and PSI light-harvesting complex, which are generally associated with xanthophylls and chlorophyll and serve as the antenna complex (Jansson, 1999). Expression of the *LHC* genes is regulated by multiple environmental and developmental cues, including light, chloroplast retrograde signal, circadian clock, abscisic acid (ABA), oxidative stress, and heat stress (Humbeck and Krupinska, 2003; Staneloni et al., 2008; Xu et al., 2012). Stresses mainly down-regulated the expression of *LHC* genes (Heddad and Adamska, 2000; Qin et al., 2008; Song et al., 2014; Kong et al., 2016), but some of the *LHC* genes were also found to be up-regulated, such as *LHCB2.1* in poplar exposed to high temperature treatment (42°C) for 6 h (Song et al., 2014). Here two *LHC* genes (*Lhca1* and *Lhcb4*) were up-regulated, and the rest are largely unchanged, indicating that the negative effects on PSII and PSI from current heat stress treatment (35°C, 6 h) were moderate. It is known that plant heat stress responses depending on both the stress intensity and duration (Schulze et al., 2005). Qin et al. (2008) reported that 21 probe sets of LHC proteins were repressed by heat particularly in the 24-h 40°C heat treatments, and pointed out that long-term heat stress damaged the photosystems more severely. In a proteomic study of alfalfa under heat stress, three chlorophyll a/b binding proteins were up-regulated at 24 h, but then decreased as the stress prolonged (72 h) (Li W. et al., 2013).

Heat stress not only affects photosynthesis by inactivating photosystems (e.g., PSII and PSI), but also by adjusting carbon fixation (Wahid et al., 2007). Here genes encoding enzymes normally involved in C4-dicarboxylic acid cycle and/or crassulacean acid metabolism (CAM) were significantly affected with two genes up-regulated (*AST* and *PEPCK*), and the other two down-regulated (*PPDK* and *NAD-ME*). This is interested since perennial ryegrass is a cool-season/C3 grass species. The PEPCK enzyme seems to be ubiquitous in plants, including C3 plant species (Leegood and Walker, 2003; Christin et al., 2009; Aldous et al., 2014). The PEPCK isoform involved in C4 photosynthesis is expressed in bundle-sheath cells, and it catalyzes the release of CO_2_ from oxaloacetate for the Calvin cycle, while AST catalyzes the reversible conversion of aspartate to oxaloacetate. In C3 plants, PEPCK also plays roles in gluconeogenesis, liberating carbon from breakdown of lipids and making the energy available for seedling growth and development (Trevanion et al., 1995; Christin et al., 2009), and in the metabolism of nitrogenous compounds in seeds (Delgado-Alvarado et al., 2007). Along with PEPCK, NAD-ME is another decarboxylation enzyme used in the inorganic carbon concentrating mechanisms of C4 and CAM plants, which oxidized malate to pyruvate and CO_2_. Non-photosynthetic isoforms of NAD-ME participates in the respiration of malate in the tricarboxylic acid cycle (Maier et al., 2011), and in *Arabidopsis* the malic enzyme has a major impact on nocturnal metabolism (Tronconi et al., 2008). PPDK participates in pyruvate metabolism and carbon fixation by catalyzing the reversible conversion of pyruvate to phosphoenolpyruvate (PEP) (Chastain et al., 2011). In C3 plants, PPDK primarily functions as an ancillary glycolytic enzyme to modulate carbon metabolism (Chastain et al., 2006; Hennen-Bierwagen et al., 2009; Taylor et al., 2010). In addition, it can also facilitate nitrogen remobilization into glutamine in senescing leaves (Taylor et al., 2010). Doubnerová and Ryšlavá (2011) suggested the functions of non-photosynthetic counterparts of C4 photosynthesis enzymes seem to be more important for plants under stresses than under optimal conditions, such as NADP-ME and PPDK. Variation of some of the genes under heat stress have been reported, and heat stress diminishes the activities of NAD(P)-ME and PPDK (Farooq et al., 2011). For instance, Wang et al. (2015) reported a reduction in PPDK activities in rice due to high temperature, which is confirmed further at both transcription and translation levels. Transcripts of AST were decreased by increased growth temperature (Duke and Doehlert, 1996). The expression changes of the four genes here might be a metabolic modulation in response to heat stress, and their exact mechanism would need further researches in future.

Plant hormones have important roles in regulating plant growth, development, and environmental stress tolerance (Davies, 2004). Under heat stress hormone homeostasis is altered, including hormone stability, biosynthesis, total contents, and compartmentalization (Maestri et al., 2002). Although the involvement of hormones in plant thermo-tolerance is complex and the exact signal pathway of hormones under heat is yet mostly unclear, many studies have shown that optimizing status of certain hormones can enhance plant thermo-tolerance (Kotak et al., 2007; Wahid et al., 2007). Cytokinins (CKs) are a group of plant hormones that play an important role in many growth and developmental processes, including promoting cell division and differentiation, enhancing chloroplast development, and counteraction of senescence (Mok and Mok, 2001). Heat stress affects CK synthesis adversely, with the reduction of CKs being reported in different species under stress, such as wild tobacco (*Nicotiana rustica*), and common bean (*Phaseolus vulgaris*) (Itai et al., 1973), winter rape (*Brassica napus* L.) (Zhou and Leul, 1999), and creeping bentgrass (Xu and Huang, 2007). Moreover, application of exogenous CKs has been shown to have effects on alleviating plant heat injury (Skogqvist, 1974; Liu and Huang, 2002; Wang et al., 2013). Adenine isopentenyl transferase (IPT) is a key enzyme catalyzing the rate-limiting step in CK biosynthesis (Sakakibara, 2006). Several studies found *SAG12-ipt* and *HSP-ipt* transgenic creeping bentgrass showed improved thermo-tolerance compared to control plants (Xu et al., 2009). Here we found *IPT* was down-regulated in perennial ryegrass under heat stress. In the meanwhile, a gene (*cis-ZOG*) involved in the *O*-glycosylation of CKs and converting active CKs into inactive or less active *O*-glucosides was up-regulated under heat stress (**Supplementary Figure S1**). All the results indicated a possible reduction of active CKs in perennial ryegrass under heat stress.

Target of rapamycin (TOR) is a Ser/Thr protein kinase that is structurally and functionally conserved among eukaryotes (yeast, plants, animals, and etc.) (Wullschleger et al., 2006). In mammals, TOR controls growth in response to growth factors (e.g., insulin), nutrients (e.g., amino acids), and cellular energy (ATP) (Loewith and Hall, 2011). And large amount of studies have been focused on the role of mTOR (mammalian TOR) in tumor development and cancer therapies (Baldo et al., 2008; Kim et al., 2016). In higher plants, TOR plays an evolutionarily conserved role in the transcription regulation of genes involved in anabolic and catabolic processes (John et al., 2011; Xiong et al., 2013). More recently, TOR was suggested to play a crucial role in modulating photosynthesis and phytohormone signaling in *Arabidopsis*, including light reaction, carbon fixation and plant hormone (auxin, ABA, brassinosteroid, and CK) signaling transduction (Dong et al., 2015; Deng et al., 2016). Here we observed an up-regulation of TOR under heat stress, which might be related to its role as a central controller of cell growth and metabolism (**Supplementary Figure S2**) (Hall, 2008).

In summary, the *de novo* assembly of perennial ryegrass transcriptome obtained more total and annotated unigenes compared to previously published ones. Most of the DEGs identified in perennial ryegrass under heat stress were relatively common to the genes reported to be responsive to heat stress in plants, including HSFs, HSPs, and antioxidant related genes. In the meanwhile, we also identified four gene candidates mainly involved in C4 carbon fixation, and one TOR gene. Their exact role in plant heat stress response would warrant further studies.

## Author Contributions

Conceived and designed the experiments: KW and WZ. Performed the experiments: YL, JT, KH, TS, and XD. Analyzed the data: YL, JT, KW, and WZ. Wrote the paper: KW. All authors reviewed and approved the final manuscript.

## Conflict of Interest Statement

The authors declare that the research was conducted in the absence of any commercial or financial relationships that could be construed as a potential conflict of interest.

## Footnotes

1 www.ncbi.nlm.nih.gov

2 www.expasy.ch/sprot

3 www.genome.jp/kegg

4 www.ncbi.nlm.nih.gov/COG

## Supplementary Material

**FIGURE S1 |** The zeatin biosynthesis pathway enriched by KEGG analysis. Gene/protein names in a red and blue box represent up-regulation and down-regulation under heat stress, respectively. EC:2.5.1.27, 2.5.1.112, adenylate dimethylallyltransferase (IPT, cytokinin synthase); EC:2.4.1.215, *cis*-zeatin *O*-glucosyltransferase (*Cis*-ZOG); EC:2.5.1.75, tRNA dimethylallyltransferase.

**FIGURE S2 |** The mTOR (mammalian target of rapamycin) signaling pathway enriched by KEGG analysis. Gene/protein names in a red and blue box represent up-regulation and down-regulation under heat stress, respectively. EC:2.7.11.1, serine/threonine-protein kinase mTOR.

**FIGURE S3 |** Linear regression analysis between Quantitative Real-Time PCR (qRT-PCR) and RNA-seq results (*r*^2^ = 0.8739) for 16 genes. *X*-axis numbers represent the fold change values of qRT-PCR results. *Y*-axis numbers represent the fold change values of RNA-seq results.

**FIGURE S4 |** qRT-PCR verification of the 16 randomly selected DEGS (differentially expressed genes). Error bars indicate standard deviation.
