# Supplementary figures and images for: Transcriptional Profiling and Identification of Heat-Responsive Genes in Perennial Ryegrass by RNA-Sequencing
Source: Front Plant Sci. 2017 Jun 21;8:1032. doi: 10.3389/fpls.2017.01032 (PMC5478880; doi:10.3389/fpls.2017.01032)

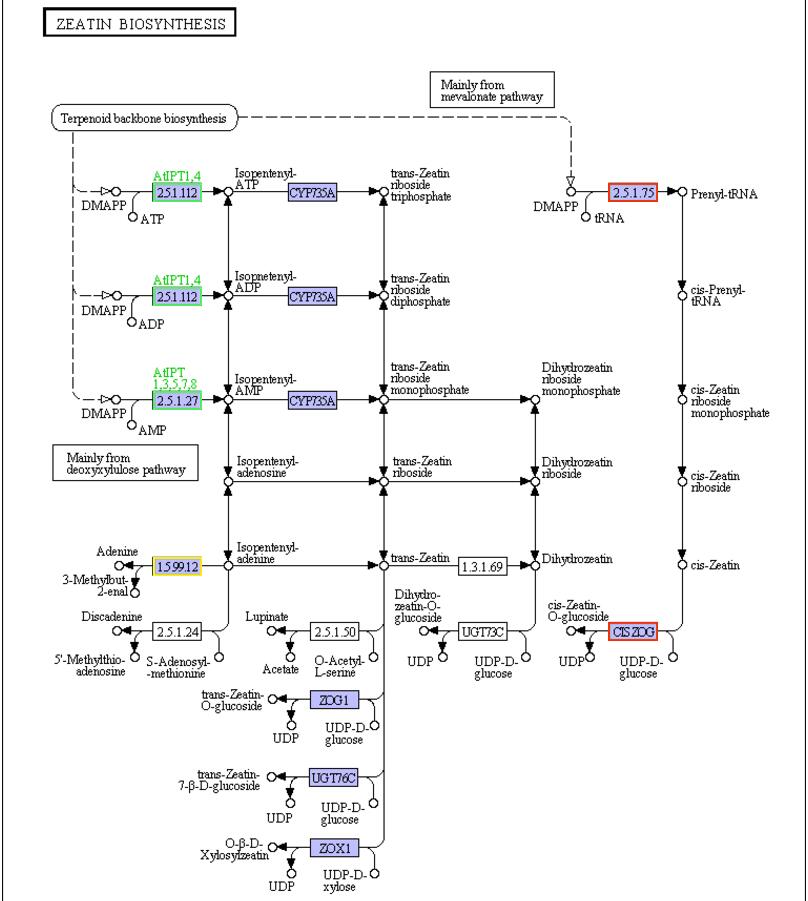

Supplement: Supplementary file 1 [file Image_1.JPEG]

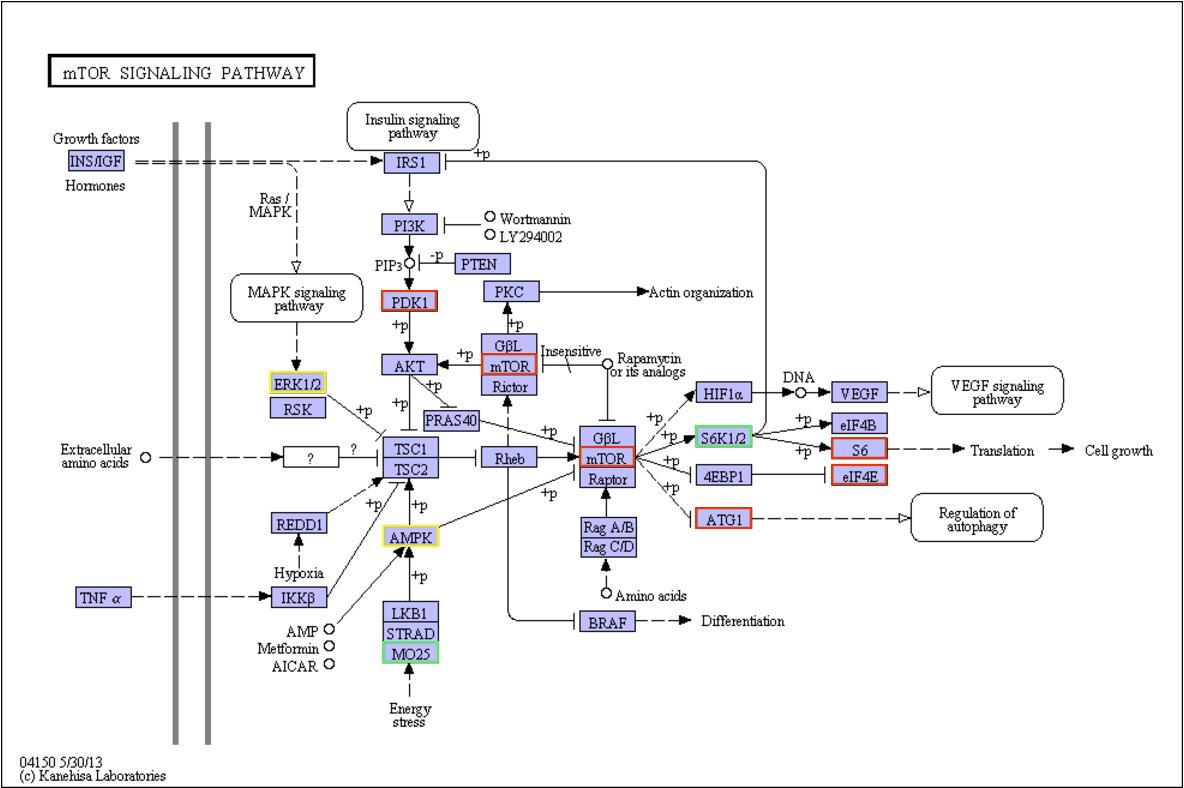

Supplement: Supplementary file 2 [file Image_2.JPEG]

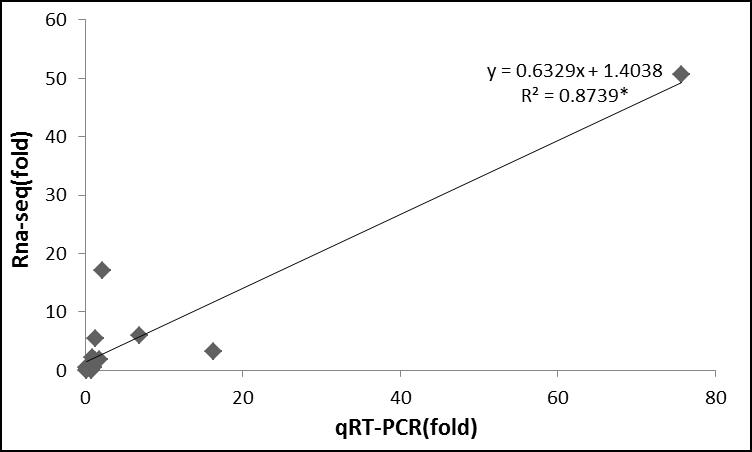

Supplement: Supplementary file 3 [file Image_3.JPEG]

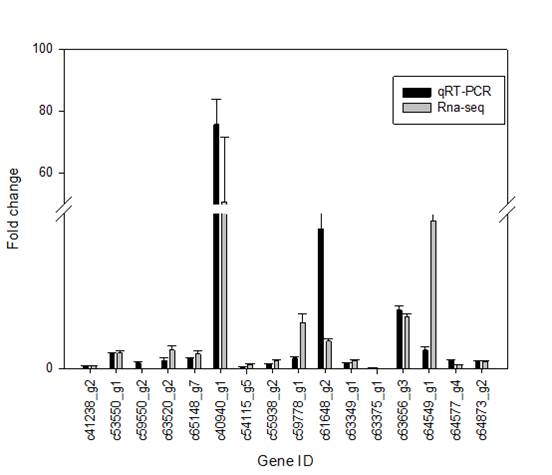

Supplement: Supplementary file 4 [file Image_4.JPEG]
